# Real-Time Optimization of Pharmacodynamic Target Attainment at Infection Site during Treatment of Post-Neurosurgical Ventriculitis Caused by Carbapenem-Resistant Gram Negatives with Ceftazidime–Avibactam-Based Regimens: A Report of Two Cases

**DOI:** 10.3390/microorganisms10010154

**Published:** 2022-01-12

**Authors:** Milo Gatti, Giulio Virgili, Pier Giorgio Cojutti, Paolo Gaibani, Matteo Conti, Carmelo Sturiale, Federico Pea, Pierluigi Viale

**Affiliations:** 1 Department of Medical and Surgical Sciences, Alma Mater Studiorum University of Bologna, 40138 Bologna, Italy; milo.gatti2@unibo.it (M.G.); pierluigi.viale@unibo.it (P.V.); 2 SSD Clinical Pharmacology, Department for Integrated Infectious Risk Management, IRCCS Azienda Ospedaliero-Universitaria di Bologna, 40138 Bologna, Italy; piergiorgio.cojutti@aosp.bo.it (P.G.C.); matteo.conti@ausl.imola.bo.it (M.C.); 3 Infectious Diseases Unit, Department for Integrated Infectious Risk Management, IRCCS Azienda Ospedaliero-Universitaria di Bologna, 40138 Bologna, Italy; giulio.virgili@aosp.bo.it; 4 Division of Microbiology, IRCCS Azienda Ospedaliero-Universitaria di Bologna, 40138 Bologna, Italy; paolo.gaibani@unibo.it; 5 Neurosurgery Unit, IRCCS Istituto delle Scienze Neurologiche Ospedale Bellaria di Bologna, 40319 Bologna, Italy; carmelo.sturiale@isnb.it

**Keywords:** ceftazidime/avibactam, CSF penetration, post-neurosurgical ventriculitis, KPC-producing *Klebsiella pneumoniae*, carbapenem-resistant *Pseudomonas aeruginosa*, fosfomycin, real-time TDM-based clinical pharmacological advice

## Abstract

We present two cases of post-neurosurgical ventriculitis caused by carbapenem-resistant Gram-negative pathogens successfully treated with high-dose ceftazidime/avibactam. The existence of a real-time clinical pharmacological advice program, by enabling the optimization of the PK/PD targets over time at the infection site, turned out to be very helpful.

## 1. Introduction

Post-neurosurgical (post-NS) infections in neurocritical patients are associated with remarkable morbidity and mortality rates [1]. In recent years, several of these infections have been found to be increasingly caused by Gram-negative pathogens with a high level of resistance against most of the available antibiotics, and this makes the implementation of appropriate treatment extremely challenging [1].

Ceftazidime/avibactam is a novel beta-lactam/beta-lactamase inhibitor combination with valuable activity against carbapenemase-resistant (CR) *Enterobacterales* and/or *Pseudomonas aeruginosa* [2]. Nowadays, data concerning ceftazidime/avibactam use in difficult-to-treat CNS infections are limited to a few case series/reports [3]. Additionally, drug exposure into cerebrospinal fluid (CSF) was only assessed at a single time-point in a patient who received systemic administration of ceftazidime/avibactam via intermittent infusion [3], and continuous infusion (CI) was shown to provide significant benefits in terms of survival rate in patients with KPC-producing *Klebsiella pneumoniae* infections treated with ceftazidime/avibactam [4].

Here, we report two cases of post-NS infections caused by CR Gram negatives that were treated with high-dose CI ceftazidime/avibactam-based regimens. Treatment was optimized by means of a real-time clinical pharmacological advice (CPA) program focused at maximizing pharmacodynamic target attainment (PD-TA) at the infection site against CR pathogens that were isolated from the CSF.

## 2. Case Presentation

**Figure 1:**
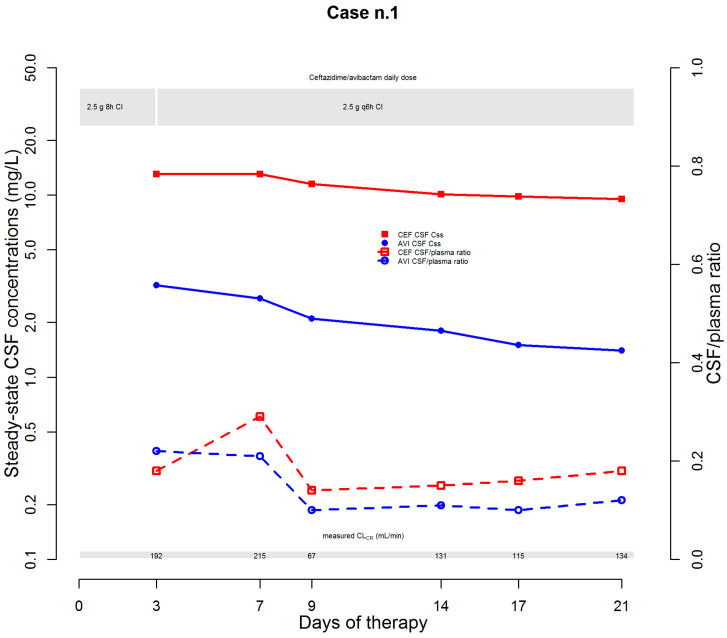
Temporal trends of steady-state CSF concentrations and of CSF/plasma ratio of ceftazidime and avibactam. AVI: avibactam; CEF: ceftazidime; CI: continuous infusion; CL_CR_: creatinine clearance; CSF: cerebrospinal fluid.

The first case was that of a 52-year-old male (who was being treated with norepinephrine, propofol, lansoprazole, enoxaparin, dexamethasone, baclofen, metoclopramide, venlafaxine, polyethylene glycol, and calcium gluconate) affected by post-traumatic cerebral ischemic injury that caused tetraplegia and hydrocephalus needing ventriculo-peritoneal shunt (VPS) positioning. During hospitalization, colonization with a CR *Klebsiella pneumoniae* (CR-KP) strain was documented at rectal swab, with susceptibility limited to ceftazidime/avibactam (MIC 4 mg/L) and colistin (MIC 0.25 mg/L). Five days later, an episode of CR-KP bacteraemia occurred, and treatment with ceftazidime/avibactam (2.5 g over 2 h loading dose (LD) followed by 2.5 g q8h over 8 h (namely by CI)) was started. CSF chemico-physical examination was carried out, and the findings were suggestive of VPS-related infection (protein 1.96 mg/dL; glucose 1.0 mg/dL; WBC count 17,695/mm^3^, 84.4% of which being polymorphonuclear cells). Consequently, VPS was replaced by an external ventricular shunt (EVD), and linezolid co-treatment was added. Soon before VPS removal and soon after EVD positioning, CSF cultures were performed. Both samples yielded CR-KP strains with the same pattern of susceptibility, and consequently, linezolid was suspended. Additionally, a real-time CPA program based on plasma and CSF therapeutic drug monitoring (TDM) was implemented for maximizing the PD-TA of ceftazidime/avibactam in both plasma and CSF over time (desired targets: steady-state concentrations (Css)/MIC ratio 4–8 for ceftazidime and 100%fT > 4 mg/L for avibactam). On day 4, both CSF pharmacodynamic targets were quasi-optimal (ceftazidime C_ss_/MIC ratio (3.25) and avibactam CSF concentration (3.2 mg/L)) (Figure 1).

Ceftazidime/avibactam dosage was increased up to 2.5 g q6h over 6 h CI. The choice was supported by the finding of an augmented renal clearance at that time (measured CLCr of 192 mL/min) and by the contention that the progressive healing of the blood–brain barrier could have led to a decreased penetration rate of ceftazidime/avibactam into CSF [5]. Subsequent TDM assessments showed CSF-to-plasma ratios ranging between 0.14 and 0.29 and between 0.10 and 0.22 for ceftazidime and avibactam, respectively. This approach allowed a quasi-optimal CSF PD-TA to be maintained over time (ceftazidime C_ss_/MIC ratio ranging 2.38–3.25; avibactam concentrations ranging 1.4–2.7 mg/L). Subsequent CSF culture assessments showed microbiological eradication and culture negativization persisted over time, with favorable clinical evolution and progressive reduction in both protein levels (from 1964 to 42 mg/dL) and WBC counts (from 17,695 to 3/mm^3^). Although ceftazidime/avibactam treatment was successfully completed on day 21, unfortunately, the patient passed away because of underlying complications of the cerebral damage.

**Figure 2:**
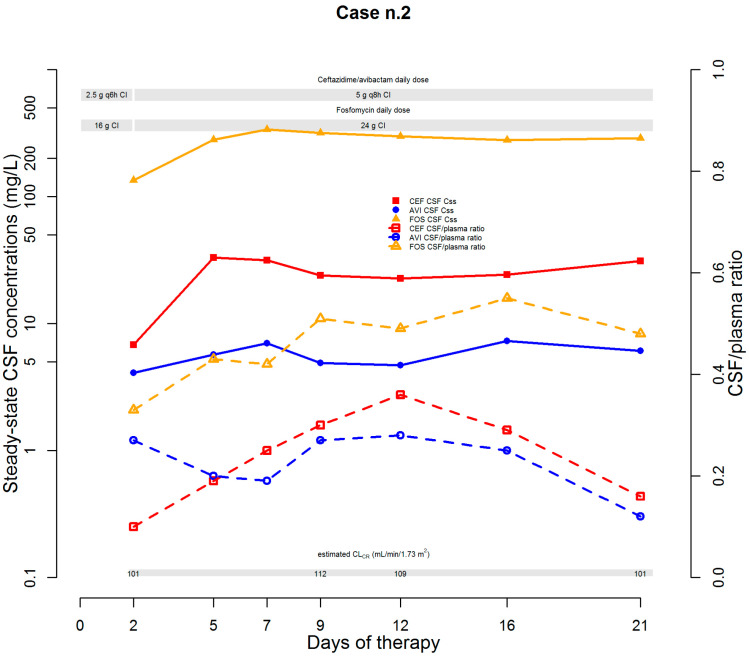
Temporal trends of steady-state CSF concentrations and of CSF/plasma ratio of ceftazidime/avibactam and fosfomycin. AVI: avibactam; CEF: ceftazidime; CI: continuous infusion; CL_CR_: creatinine clearance; CSF: cerebrospinal fluid; FOS: fosfomycin.

The second case was that of a 52-year-old male (who was being treated with lansoprazole, sertraline, levetiracetam, delorazepam, and enoxaparin) affected by a pineal neoplasm complicated by obstructive hydrocephalus. After neurosurgical exeresis, an EVS was positioned, and the post-NS course was complicated by fever coupled with headache relapse. CSF chemico-physical examination was suggestive of an EVS infection (protein 122.0 mg/dL; WBCs 176/mm^3^, 65.9% of which being polymorphonuclear cells). Empirical treatment with cefepime plus trimethoprim-sulfamethoxazole was started. On day 3, CSF culture was positive for a CR-*Pseudomonas aeruginosa* strain. The strain was susceptible to ceftazidime/avibactam (MIC 4 mg/L), amikacin (MIC 8 mg/L), and ceftolozane/tazobactam (MIC 1 mg/L). Testing by agar dilution showed an MIC for fosfomycin of 64 mg/L. After EVS removal and substitution, antibiotic therapy was consequently switched to ceftazidime/avibactam (2.5 g q6h over 6 h CI after 2.5 LD) plus fosfomycin (8 g LD followed by 16 g q24h CI). A real-time CPA program was implemented for maximizing the PD-TAs of both ceftazidime/avibactam and fosfomycin into CSF (desired targets: for ceftazidime/avibactam, as previously mentioned; for fosfomycin, *f*AUC/MIC ratio ≥ 40.8 [6]). On day 2, CSF PD-TAs were mixed for ceftazidime/avibactam (ceftazidime C_ss_/MIC ratio 1.7, avibactam concentration 4.1 mg/L) and borderline for fosfomycin (*f*AUC/MIC ratio 50.3 (Figure 2)).

Estimated CLCr was 101 mL/min/1.73 m^2^, and dosages of both antibiotics were increased (up to 5 g q8h over 8 h CI for ceftazidime/avibactam and 24 g q24h CI for fosfomycin). This choice was also supported by the contention that the progressive healing of the blood–brain barrier could have led to a decrease in the penetration rate of both antibiotics into CSF [5]. Subsequent CSF assessments showed a CSF-to-plasma ratio ranging between 0.16 and 0.36 and between 0.12 and 0.28 for ceftazidime and avibactam, respectively. PD-TAs of both ceftazidime and avibactam were always optimal (ceftazidime C_ss_/MIC ratio ranging from 5.675 to 8.25 and avibactam concentrations ranging from 4.7 to 7.3 mg/L). For fosfomycin, CSF-to-plasma ratios ranged between 0.42 and 0.5, and the CSF PD-TAs were always optimal as well (*f*AUC/MIC ratio range 104.63–126.38). Subsequent CSF assessments showed microbiological eradication and culture negativization persisted over time, with favorable clinical evolution and progressive reductions in both protein levels and WBC counts. EVS was removed on day 23 and antibiotic therapy was stopped on day 26 after achieving optimal clinical response. The patient was discharged to a rehabilitation facility, and no relapse at follow-up occurred.

## 3. Discussion

Overall, these two cases firstly showed that the CSF penetration rates of ceftazidime and avibactam in patients with post-NS infections ranged over time around 15–30% during the first 3 weeks post-event. Additionally, our findings showed that optimizing CSF PD-TAs by means of a real-time CPA program may be valuable in tackling CR-related post-NS infections caused by borderline susceptible pathogens. Repeating TDM assessments over time may also be crucial in neurosurgical patients. This approach may allow the posology to be modulated day-by-day according to the dynamic evolution of the CNS infection [5] and/or to the fluctuations of renal function, given that ARC is a highly probable occurrence in the early post-event phase of neurocritical patients [7]. Furthermore, it may also concur in avoiding the need for intrathecal administration of antibiotics that could be complicated by potential CNS adverse events. Notably, in the patient who experienced ARC, treatment with ceftazidime/avibactam doses up to 10–15 g/day did not cause any adverse event. Finally, it should be mentioned that the choice of combining ceftazidime/avibactam with fosfomycin for treating CR-*P. aeruginosa* related post-NS infection was based on preclinical studies showing synergism. In a murine infection model using a high bacterial burden, the combination of ceftazidime/avibactam with fosfomycin was superior to either drug alone in significantly reducing the *P. aeruginosa* burden [8]. Notably, the high CSF penetration rate of fosfomycin (45–50%) allowed optimal PD-TAs over time in our patient by administering doses up to 24 g q24h CI.

In conclusion, our findings showed that the existence of a real-time CPA program, by enabling optimization of the PK/PD targets over time at the infection site, turned out to be very helpful in granting successful treatment with high-dose CI ceftazidime/avibactam-based regimens of two cases of post-NS ventriculitis caused by CR-Gram-negative pathogens. Of course, ours is simply a proof-of-concept and we are aware that further prospective confirmatory studies are warranted before any definitive conclusion can be drawn.

## Data Availability

The data presented in this study are available on request from the corresponding author. The data are not publicly available due to privacy concerns.
